# Access to HIV services and viral load suppression among children during the 90-90-90 strategy implementation in South Africa: A time series analysis

**DOI:** 10.4102/sajhivmed.v22i1.1187

**Published:** 2021-03-17

**Authors:** Juliet C.Y. Nyasulu, Innocent Maposa, Bernard P. Sikhakhane, Himani Pandya

**Affiliations:** 1 Division of Community Paediatrics, Faculty of Health Sciences, University of the Witwatersrand, Johannesburg, South Africa; 2 Health Systems Strengthening, AFRIQUIP, Johannesburg, South Africa; 3 Department of Epidemiology and Biostatistics, Faculty of Health Sciences, University of the Witwatersrand, Johannesburg, South Africa; 4 Centre for HIV and STI’s, National Institute for Communicable Diseases, Johannesburg, South Africa; 5 JHB Health District: Monitoring and Evaluation, Gauteng Provincial Department of Health, Johannesburg, South Africa

**Keywords:** 90-90-90 strategy, implementation, HIV testing, ART initiation, retention in HIV care, HIV care access by children

## Abstract

**Background:**

During the era of the Millennium Development Goals (MDG), children were shown to have less access to human immunodeficiency virus (HIV) services than their adult counterparts; hence the call to prioritise children in the implementation of the Sustainable Development Goals (SDGs). However, South African (SA) national data in 2019 indicated that almost 3 years into the implementation of the 90-90-90 strategy, only 59% of children living with HIV had been tested for HIV compared to 90% of adults.

**Objectives:**

To evaluate the access of children to HIV services and record the viral load (VL) suppression rates during the implementation of the 90-90-90 strategy in the City of Johannesburg (COJ), South Africa.

**Methods:**

This study applied a quasi-experimental interrupted time-series (ITS) design using the monthly District Health Information System (DHIS) and National Health Laboratory Services (NHLS) databases spanning the period from 2015 to 2020, that is, before and after the implementation and roll-out of the 90-90-90 strategy. Data were extracted from these databases into MS Excel 2010 spreadsheets and analysed with Stata 15 software from Stata Corp using a two-tailed *t*-test at a 5% level of significance.

**Results:**

Overall, a significant increase was observed in the number of individuals tested for HIV, *n* = 757, *p* = 0.0086, and retained in care *n* = 2523, *p* = 0.001 over the whole period of analysis beginning in April 2015. Adult HIV testing, antiretroviral treatment (ART) initiation and retention in care had been decreasing in absolute numbers over a 10-month period before the intervention. An increase in these three data elements was observed following the implementation of the 90-90-90 program. On the other hand, children aged 0–15 years had demonstrated a significant increase in absolute numbers tested for HIV, *n* = 171, *p* = 0.001, but an insignificant increase in number of ART initiations, *n* = 14.33, *p* = 0.252, before implementation but a decrease after this. The overall VL suppression rates for children were lower than those of adults.

**Conclusion:**

Although the COJ has recorded progress in adult HIV testing, ART initiation and retention, children living with HIV aged 0–15 years continue to experience less access to HIV services and lower VL suppression than youths and adults of ≥ 15 years. Therefore, to ensure that the 90-90-90 targets are achieved across different age groups, children must be prioritised so that they can equally access these services with adults.

## Introduction

In the era of the Sustainable Development Goals (SDG’s), the UNAIDS set countries, the ambitious ‘90-90-90’ target of eradicating global infection with HIV by 2030. This required that by 2020, 90% of people living with HIV would know their status, 90% of whom would be on antiretroviral treatment (ART) and 90% of the latter, would be virally suppressed.^1^ The underlying principle behind these goals is the rapid scale-up of access to treatment for all PLWH, irrespective of their CD4 count. The primary target are cities as the risk of HIV and tuberculosis (TB) is greater in urban than rural areas. More than 200 cities and municipalities across the globe signed the United Nations declaration pledging commitment to these goals.^1,2^ Some progress was made: by 2018, 79% (range, 67–92%) of global PLWH knew their status. Of these, 78% (range 69–82%) were accessing ART and 86% (range, 72–92%) of the latter were virally suppressed. Targets for children and adolescents lagged behind.^1^

A total of 7.9 million South Africans are PLWH. Of these, ± 4.9 million are on ART.^3^ The City of Johannesburg (COJ), together with 19 other South African (SA) cities and metropolitans, endorsed these 90-90-90 strategic goals.^1,2^ Whilst testing and treatment ‘scale-up’ in the COJ had been rapid in the preceding decade, the required ‘doubling of effort’ by 2020, could not be met.^4^ Nonetheless, progress had been made. Ninety per cent of SA-PLWH knew their status, 62% had been initiated on ART and 54% were virally suppressed, that is, 90-62-54%.^3,5^ All was not good news, though. In 2019, only 63% of 0–15-year-olds living with HIV were reported to be on ART.^5^ Compared with adults, HIV testing of children < 15 years has been suboptimal. The 2019 SA-90-90-90 progress report indicated that only 59% of children living with HIV knew their status, and though 96% of those tested had been initiated onto ART, only 67% were virally suppressed; too few children are tested.^6^ This is despite the 2016–2030 SDG’s strategy to prioritise children and the young.^7,8^ Although the national HIV statistics show a decline in the number of infected children and an increase in adults with HIV, the extent of the problem in the COJ has not been reported.^3^ This study evaluates the access of children < 15 years to HIV services and the rates of viral load (VL) suppression during the implementation of the 90-90-90 strategy in the city.

## Methodology

### Study design

This was a quasi-experimental study that aimed to establish the impact of the implementation of the 90-90-90 strategy on HIV testing, ART initiation, retention in care and viral suppression amongst PLWH in the COJ using an interrupted time series (ITS) analysis of data from the District Health Information System (DHIS) and the National Health Laboratory Service (NHLS). To strengthen the internal validity of the study, other factors that would impact HIV testing, ART usage and retention in care during the study period are explored and explained.

### Study participants

The study included all individuals who tested positive for HIV and were initiated onto ART and who remained on ART in the COJ and on the DHIS database over the study period. In line with the DHIS age-categories, children were grouped into those < 59 months of age and from 5 to < 15 years. Those above 15 years were categorised as adults.

All VL tests performed by the NHLS during the study period were included.

#### Study setting

Data were collected from the COJ, Gauteng Province, South Africa. The city has seven regions (A–F), and a total population of ± 5.6 million. This accounts for 36% of the Gauteng and ± 10% of the national population.^3^

One hundred and twenty-one primary healthcare (PHC) facilities offer HIV-services in the COJ and cater for ± half-a-million PLWH.^9^

### The 90-90-90 strategy intervention

The 90-90-90 strategy was conceptualised in 2015 and introduced to the COJ in January 2016. The district program implementation team identified indicators that would assist in attaining these targets. These indicators focused on the four pillars of HIV-care cascade, namely (1) prevention, (2) case identification, (3) treatment initiation, and (4) retention and success of treatment. Those indicators performing below target were described together with their root causes. Activities were proposed to address the gaps in the 90-90-90 district strategy implementation plan (DIP).^7^ Responsible personnel and the measures required to track progress were identified.

For example, one of the gaps in the district was poor access to services by youth and by men. As a result, social mobilisation and communication that targeted men and youth, key populations and vulnerable groups was implemented. The DIP was rolled out in January 2016.^7^

### Data collection

Data were collected from monthly reports of facilities captured in the DHIS database. All who were initiated on ART had a VL test – a measure of viral suppression and a proxy of adherence and retention in care – after 6 months and annually thereafter. More frequent VL monitoring is recommended where VL suppression is not achieved.^10^ Viral suppression is defined as VL < 50 copies/mL as per the 2019 South African National Department of Health (SANDoH) guidelines.^10^ The NHLS database provided viral suppression data. Data from the DHIS program was collated from April 2015 until March 2019 (48 months) and included the 9 months to January 2016 (*before*) and the 38 months *after* strategy implementation in January 2016. In addition, all VL suppression NHLS data from January 2015 to February 2020 (62 months) are included in this study. The proportion of virally suppressed ‘test-results’ has been calculated on a monthly basis throughout the 62 months for both adults and children.

### Outcome measures

The study outcome measures included the number of individuals tested, initiated on ART, retained in care and virally suppressed in the COJ facilities before and after the January 2016 roll-out.

### Statistical analysis

Data preparation was done in MS Excel 2010 and analysed with Stata 15(Stata Corp). We used a two-tailed test at a 5% level of significance throughout the analysis. Continuous data were expressed as the means (standard deviation, SD) or medians (interquartile range, IQR). Categorical data are presented as frequencies and percentages. The Durbin-Watson statistic was used to test for autocorrelation. We modelled the ITS data using segmented regression analysis which, as suggested by Wagner et al., has the advantage of adjusting for baseline level and trend, in this case, the HIV test-and-treat policy changes.^11^ For HIV testing, ART initiation and retention in HIV care, we compared the time series pattern 9 months before and after January 2016 when the 90-90-90 strategy implementation was rolled out in the COJ to assess differences. In addition, average monthly VL suppression rates from 12 months (January–December 2015) before were compared to 50 months (January 2016–February 2020) after strategy implementation roll-out. The trends before and after this intervention were analysed to assess whether the passage of time was associated with an increase or a decrease in the number of HIV tests, initiations and retention in care as well as viral suppression.

The specific model is: $Y_{t}=\beta_{0}+\beta_{1}X_{t}+\beta_{2}(I(t\geq t_{0}))+\beta_{3}X_{t-t_{0}}+e_{t}$ where *Y_t_* is the total number of those HIV tested, ART initiated and remaining in care or VL suppression rate in a month in Johannesburg, which are response variables in this model; *X*_*t*_ is a count time variable indicating time in months from the start of the observation period to intervention; intervention is an indicator *I(t* ≥ *t*_0_*)* variable for time *t* occurring before (*I(t* ≥ *t*_0_*)* =0) or after (*I(t* ≥ *t*_0_*)* = 1) 90-90-90 strategy roll-out, which was implemented at month *t*_0_ in the series; and $X_{t-t_{0}}$ representing time after intervention is a count variable for the number of months after the intervention at time *t*_0_ = 10 when assessing 90-90-90 indicators and *t*_0_ = 13 when assessing suppression rates computed from NHLS data. Suppression was defined as having a VL below 50 copies/mL.

In this model, *β*_0_ is the estimate of baseline level of the outcome, being the average number of patients tested for HIV, initiated on ART and virally suppressed at time zero; *β*_1_ estimates the mean change in total number or rate that occurred in each month before 90-90-90 strategy intervention; *β*_2_ estimates the level of change in the average number of patients tested for HIV, initiated on ART and virally suppressed during the intervention month; and *β*_3_ estimates the change in the trend in the mean monthly number of patients tested for HIV, initiated on ART and virally suppressed after the 90-90-90 strategy intervention implementation. The error term *e*_*t*_ at time *t* represents the random variability not accounted for by the model.

### Ethical considerations

Ethical approval for this study was obtained on 01 October 2018 from the University of the Witwatersrand Human Research Ethics Committee (Project research number: R14/49 and ethical clearance number: M180640).

## Results

Numbers for HIV testing, ART initiation and those remaining in care before and after the implementation roll-out.

### Pre-intervention or pre-implementation

All values are presented as medians with interquartile range [IQR].

**The total number tested monthly for** HIV in the pre-intervention period viz. April–December 2015 was a median of *n* = 64 379 (IQR, 58 425–66 374). Adults numbered 49 650 (IQR, 47 644–52 092) of whom 6592 (IQR, 6328–6808) were in antenatal care (ANC). Under-5 years numbered 544 (IQR: 0–786) and 5 to < 15-year-olds numbered 681 (IQR, 25–917).

Overall, **the total number initiated monthly onto ART** in the pre-intervention period was 4431 (IQR: 4375–4513). *N* = 101 (IQR, 89–112) were children < 15 years. The number of adults was 4327 (IQR: 425–4388). The **total number of PLWH retained in antiretroviral (ART) care monthly** in the pre-intervention period was 256 278 (IQR: 254 111–257 452). *N* = 10 775 (IQR, 10 704–10 832) were children < 15 years of age. Youths and adults in care numbered 245 509 (IQR: 243 392–246 620).

### Post-intervention or post-implementation

All values are presented as medians with interquartile ranges [IQR].

The **total number tested monthly for HIV** during the 38 months post-intervention period was 75 162 (IQR: 71 015–84 736). Youths and adults numbered 57 027 (IQR, 51 831–64 825) of whom 7085 (IQR, 6725–7461) were in ANC care. The number of under-5 years was 1065 (IQR: 893–1194) and 5 to < 15 years was 1449 (IQR, 1174–2097) monthly.

The **total number initiated monthly onto ART** post-intervention was 5288 (IQR, 4796–6053). *N* = 129 (IQR: 103–173) were children < 15 years. The youths and adults numbered 5134 (IQR: 4659–5925).

The **total number of PLWH retained on ART monthly** in the 38 months after intervention was 312 360 (IQR, 284 296–338 008). The monthly numbers of children < 15 years were 8688 (IQR, 8266–10 048). The median monthly number of all youths and adults retained in care was 303 596 (IQR, 274 060–329 767).

### Number of viral load tests conducted before and after intervention

A total of 2 040 018 VL tests were performed during the 62-month (5 years) period of the study on 23 193 children < 5 years, 58 563 children 5 to < 15 years and 1 958 262 youths and adults (Table 1). Each newly diagnosed client had a VL at 6 and 12 months then annually thereafter and intermittently if clinically indicated. Follow-up tests number more than the total number of individual patients.

**TABLE 1:** Number of viral loads captured annually in the City of Johannesburg before and after the intervention.

Age category	Pre-intervention (ITV): 2015 (12 months)	2016 (post-ITV)	2017 (post-ITV)	2018 (post-ITV)	2019 (post-ITV)	2020 (post-ITV) Jan/Feb (2 months)	Total
< 5 years	4575	4561	4667	4538	4191	661	23 193
5–15 years	10 956	11 974	11 583	11 060	11 259	1731	58 563
Adults	264 690	341 270	396 448	420 527	455 541	79 786	1 958 262
Overall tests	280 221	357 805	412 698	436 125	470 991	82 178	2 040 018

ITV, intervention.

#### HIV testing, antiretroviral treatment initiation and retention in care before and after the intervention

### HIV testing before and after the intervention

In the 10 months of 2015 that preceded the implementation of the 90-90-90 roll-out strategy, there was a non-significant monthly average decline in the number tested for HIV, *p* = 0.57. However, categorised by age, HIV testing of adults did appear to decline significantly, *p* < 0.001; whilst testing of children increased, 19 months to < 5 years, *p* = 0.008 and ≥ 5 to < 15 years, *p* = 0.001 (Table 2, Item 1). After controlling for autocorrelation, at the time of the roll-out-strategy intervention in January 2016 (month 10) a non-significant, monthly-average increase of 1448 people tested for HIV, *p* = 0.27 was recorded. Categorised by age, this testing showed a significant increase amongst adults (*p* < 0.001) and a significant decrease amongst children, 19 to < 5 years, *p* = 0.008, and those 5 to < 15 years, *p* = 0.001. Nevertheless, in the period 2015–2019, a significant overall average increase of 757 persons was recorded in the monthly average tested for HIV, *p* = 0.009.

**TABLE 2:** The monthly average change in HIV testing and ART initiation of youths and adults (> 15yrs) and of children (< 15yrs) before (9 months) and after (38 months) the 90-90-90 implementation or intervention.

Variables	Before 90-90-90	*p*	After 90-90-90	*p*	Overal trend	*p*
**All HIV tests before and after intervention**						
Parameter	$\hat{\beta_{1}}$	-	$\hat{\beta_{3}}$	-	$\hat{\beta_{3}}+\hat{\beta_{1}}$	-
Monthly average	−671	< 0.001*	1448	0.270	757	0.0086*
95% CI	[53230.04; 76349.15]	-	[-1163.94; 4059.80]	-	[202.05; 1311.08]	-
**Average monthly increase or decrease in HIV testing among adults, ANC and children**						
Parameter	$\hat{\beta_{1}}$	-	$\hat{\beta_{3}}$	-	$\hat{\beta_{3}}+\hat{\beta_{1}}$	-
Adults	−948	0.001*	1570	0.180	622	0.0250*
ANC	−27	0.740	32	0.700	5	0.5300
19 to < 59 months	115	0.008*	−101	0.021*	141	0.0020*
5 to < 15 years	171	0.001*	−171	0.001*	0.30	0.9900
**Antiretroviral initiation (ART start)**						
Parameter	$\hat{\beta_{1}}$	-	$\hat{\beta_{3}}$	-	$\hat{\beta_{3}}+\hat{\beta_{1}}$	-
Monthly average	−52	0.510	59	0.484	7	0.3070
95% CI	[53230.04; 76349.15]	-	[-1163.94; 4059.80]	-	[-36.94; 50.19]	-
**Average monthly increase or decrease in ART initiation**						
Parameter	$\hat{\beta_{1}}$	-	$\hat{\beta_{3}}$	-	$\hat{\beta_{3}}+\hat{\beta_{1}}$	-
Adults (> 15 years)	−64.59	0.349	74.15	0.307	9.57	0.6090
< 15 years	14.33	0.252	−16.73	0.245	−2.41	0.4000
**Retention on ART among adults, and childen**						
Parameter	$\hat{\beta_{1}}$	-	$\hat{\beta_{3}}$	-	$\hat{\beta_{3}}+\hat{\beta_{1}}$	-
Monthly average	−462	0.510	2985	< 0.001*	2523	< 0.0010*
95% CI	[-1879.45; 956.25]	-	[1503.01; 4466.48]	-	[2288.66; 2757.64]	-
**Monthly increase or decrease for those retained on ART**						
Parameter	$\hat{\beta_{1}}$	-	$\hat{\beta_{3}}$	-	$\hat{\beta_{3}}+\hat{\beta_{1}}$	-
Adults (> 15 years)	−418.34	0.536	3014.74	< 0.001*	2596.40	< 0.0010*
< 15 years	48.52	0.320	−23.97	0.615	−72.49	< 0.0010*

Note: *, Shows significant differences in the parameters before and after the strategy implementation.

HIV, human immunodeficiency virus; ART, antiretroviral treatment; ANC, antenatal care; VL, viral load.

#### Post-intervention or post-implementation

A non-significant increase in HIV testing was observed in the total adult group and the ANC attendees, though not significantly different from the baseline. On the other hand, significant declines in HIV testing amongst under-5 years (< 59 months) and 5 to < 15 year groups were observed at the same time: *n* = -101; *p* = 0.021 and *n* = -171; *p* < 0.001, respectively.

#### Overall trend throughout the study period

As shown in Table 2, overall, there was a significant increase in HIV testing amongst adults and under-5 years children (< 59 months): *n* = 622; *p* = 0.025 and *n* = 14; *p* = 0.002, respectively, with no significant change from baseline for the 5 to < 15-year-old group.

### Antiretroviral treatment initiation for those who are HIV positive

Pre-implementation, the average numbers of monthly ART initiations amongst adults declined non-significantly by about 65 persons, *p* = 0.349, but increased by ± 14 persons amongst children < 15 years, *p* = 0.252. *After implementation*, an increase was observed in adults and a slight decrease in children < 15 years.

Overall, the trend throughout the study period suggests an insignificant increase in ART initiation amongst adults and an insignificant decrease amongst children < 15 years.

### Retention on antiretroviral treatment

For 9 months before implementation, there was a non-significant total monthly average loss from HIV care of 462 PLWH, *p* = 0.515. After January 2016, at the time of the strategy intervention, after controlling for autocorrelation, there was a monthly average increase of retention in care of 2985 PLWH, *p* < 0.001. Overall, from 2015 to 2018, there was a significant monthly average increase of 2523 PLWH retained in ART care, *p* < 0.001.

#### Pre-intervention or pre-implementation

As shown in Table 2, the average number of adults in ART care declined by about 418 persons, *p* = 0.536, monthly and in children (the < 15-year group) by ± 49 persons monthly, *p* = 0.302 (Table 2).

#### Post-intervention or post-implementation

There was an increase in adults and a decrease in children < 15 years retained on ART care (Table 2).

#### Overall trend throughout the study period

There was a significant increase in numbers of adults on ART and a significant decrease in children < 15 years.

### Overall HIV testing and antiretroviral treatment initiation trends over the study period

**FIGURE 1:**
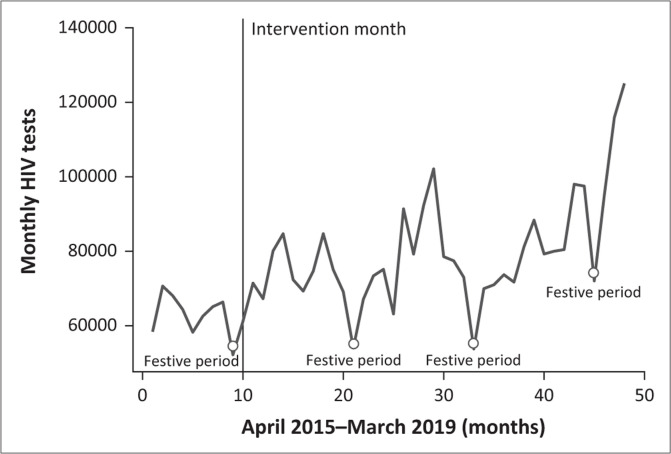
Overall average monthly increase or decrease in human immunodeficiency virus testing in the City of Johannesburg.

Figure 1 shows the overall increasing monthly HIV testing trends over the study period and the consistent declines in update of HIV testing over the festive months of December and January.

**FIGURE 2:**
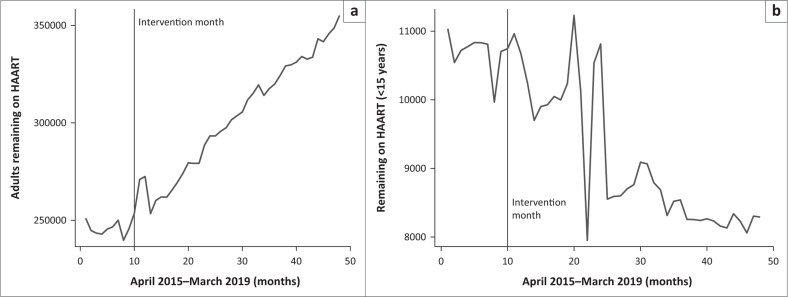
(a) Trends in adults monthly retention on antiretroviral treatment and (b) trends in < 15 years monthly retention on antiretroviral treatment.

Figure 2a and 2b depicts the increasing numbers of those retained on ART amongst adults centrally to a continuous drop amongst children over the months. To note is the fact that adult and children retention in HIV care trends had some consistent drops during the festive season months of December to January.

### Viral load suppression rates before and after the strategy roll-out

**FIGURE 3:**
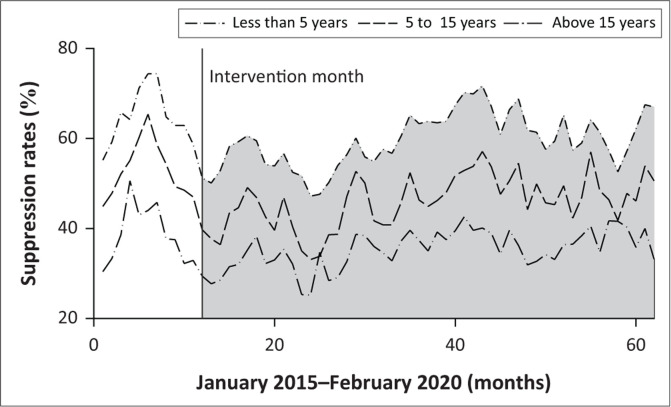
Proportions of those virally suppressed before and after the 90-90-90 strategy implementation.

As shown in Figure 3, the number of VL tests conducted during January 2015 to February 2020 indicates a higher suppression rate for adults compared to children through the 5 years pre- and post-implementation and roll-out. Throughout the period, a seasonal drop in VL suppression is observed between December and January of each year.

## Discussion

This was a quasi-experimental study that utilised an ITS analysis to establish the effect of the 90-90-90 strategy implementation on HIV testing, ART initiation and retention in care amongst HIV positive individuals in the COJ. Overall, a significant increase was observed in the number of individuals tested for HIV, initiated on ART and retained in HIV care after the implementation. Adult HIV testing, ART initiation and retention in HIV care had decreased significantly before implementation but was followed by a significant increase over time in all three aspects once the strategy was rolled out. On the other hand, all three measures in children aged 0–15 years had been increasing before implementation but decreased significantly thereafter.

Throughout the study period, rates of viral suppression were less in children than in adults, though trends were similar (Table 3). Rates of HIV testing, ART initiation, retention in care and VL suppression declined in December and January – each ‘festive season’ – throughout the study period.

**TABLE 3:** Viral load suppression rate before and after the 90-90-90 strategy implementation amongst children and adults. Viral load defined at VL< 50 copies/mL.

Intervention trends	Parameter	Adults	Under 5 years	5–15 years
Before 90-90-90 strategy	$\hat{\beta_{1}}$	−0.32	−0.009	0.04
*p*		0.645	0.990	0.953
Change at intervention	$\hat{\beta_{2}}$	−9.1	−6%	−10
*p*		0.002	0.1	0.002
After 90-90-90 strategy	$\hat{\beta_{3}}$	−0.1	+0.15	+0.15
*p*		0.900	0.800	0.800
Overall trend	$\hat{\beta_{3}}+\hat{\beta_{1}}$	0.202	0.139	0.187
*p*		0.080	0.014	0.033

Note: A drop in the VL suppression rate was observed over the 12-month observation period before the intervention in both children and adults in 2015. This drop was significant amongst adults and children aged 5–15 years. This was followed by a non-significant and slow rise in children’s viral suppression rates and a drop amongst adults. Overall, only children aged < 5 years and 5 to < 15 years showed a marginal but significant increase in VL suppression over the 5-year study period.

### HIV testing, antiretroviral treatment initiation and retention in care for adults

Our data indicate that the implementation of the 90-90-09 strategy in the COJ has had a positive effect amongst adults accessing HIV services in the city. Other studies support this assessment.^12,13^ The roll-out of ‘Universal Test and Treat’ (UTT) in September 2016 would have contributed to this outcome as this removed the CD4 count ‘barrier’ to care.^14,15^ Furthermore, the roll-out of the 90-90-90 strategy was accompanied by HIV testing campaigns that raised awareness in the community.^7^

### HIV testing, antiretroviral treatment initiation and retention in care for 0–15 year-olds

A concern is the apparent decline of HIV testing amongst children aged 0–15 years. This followed an increase in testing in the pre-implementation phase. The most recent SANDoH implementation progress-report noted that only ± 59% of children living with HIV (CLWH) are being diagnosed. Although 96% of those who test positive are initiated onto ART, only 66% are retained in care. In reality, this means that only about 38% of CLWH achieve retention on ART.^6^

Furthermore, the HIV screening strategy amongst the 0–15 year-olds during the study period missed a large number of CLWH, especially children infected postnatally via breastfeeding practices, or perinatally with delayed seroconversion post-delivery.^16^ Birth PCR tests on all exposed infants are now helping to close this gap.^17,18^ Additional failures occur during or at the end of breastfeeding, or when birth results are not checked and when follow-up and linkage to care are not established.^19^ Point-of-care infant testing may assist in the prevention of some gaps.^20^ Current South African infant-testing guidelines recommend testing at birth, 10 weeks and upon ending breastfeeding.^14^ Nonetheless, data from Tshwane district report that only a third of HIV-exposed children repeat the HIV test at 18–24 months and only a quarter at the end of breastfeeding.^21^ Although early infant diagnosis using PCR at birth and 10 weeks has impacted infant HIV testing,^22^ gaps remain. Consequently, the current testing of children is suboptimal compared to adults.^6^ The system is failing children. Greater effort must be made to trace and test exposed children, especially post-breastfeeding as these 18-month rates are extremely low.^6,23^

Our study found that ART initiation rates in < 5-year-old children have declined. In part, this results from the country’s excellent prevention of mother-to-child transmission of HIV (PMTCT) rate of 0.9% and the COJ’s 1%.^24^ Fewer children are now needing to be initiated on ART. However, our study also found that fewer children are being tested. Gaps and opportunities are being missed.^6^ Innovative approaches are still needed in support of this population group.^20,25^

The testing, initiation and retention in care data of the 5 to < 15-year-old cohort changed from an increase in 2015 to a significant decline after the implementation of the 90-90-90 strategy. A possible explanation is that at the end of the implementation phase of the Millennium Development Goals (MDG) goals (2014–2015), it was clear that children were being missed and a special call was made to reverse this situation with the implementation of the SDGs. A concerted effort was made to prioritise HIV testing and ART initiation in this age group.^8^ Although the COJ had a 2016–2020 DIP which covered activities to reach youths, it did not prioritise those aged < 15 years.^8,26^ Reports in the SDG implementation era still document the inadequate provision of services to children and youth; something that must be rectified in the 2020/2030 targets.^13,23^

Concerning children and youths, the NHLS database indicated that throughout the reporting period, the younger the individual, the lower the VL suppression rate. Whilst children aged < 5 years and 5 to < 15 years showed a small but significant increase in the overall VL suppression over the 5-year study period, this was still below that of the adults. The suboptimal performance of children triangulates with the DHIS database and is similar to other studies.^6^ Innovative solutions are needed to assist in ensuring that more children are tested, initiated and adhere to ART. Five years into the implementation of SDG goals, our children lag behind their adult counterparts. Urgent action is called for.

Of interest is the ‘lapse’ in the performance indicators across all age groups in the December to January festive period. Not much has been documented regarding patients’ access and adherence to care during this time.

However, we speculate that the lower rates of performance at these times reflect the annual migration of city-dwellers to ancestral homes in South and Southern Africa – appointments are missed and treatment adherence impaired and VL suppression rates fall.^3^ It is critical to establish whether this is the case and what mechanisms are needed to mitigate the problem. For example, the provision of more than one repeat of medication to cover the holiday duration. And the scheduling of the COJ’s HIV testing campaigns to avoid the holiday seasons.

Study limitations include the retrospective nature of the data and our inability therefore to link cause and effect.

Caution is suggested when interpreting sensitivity data where numbers of our children analysed were extremely low. The study also records that the COJ was not able to fulfil the UNAIDS 90-90-90 target by 2020. The databases and data recorded in healthcare facilities were at times incomplete. However, we applied quality tracing to our resources and are confident that the outcome in this study reflects that data. The strength of the study is in the large cohort size and the reproducibility and integrity of the data over a prolonged 5-year assessment period.

## Conclusion

This study highlights the suboptimal level of access to HIV services and VL suppression of children compared to adults during the 90-90-90 strategy implementation in the COJ. Serious consideration is recommended to ensure that children and their guardians enjoy equity with adults in accessing healthcare services in the COJ if 90-90-90 targets are to be met. Plans must be implemented to mitigate the loss of HIV care and control that appears to be occurring during the holiday season.
